# Establishing a scholarly culture requires a conceptual framework for leveraging change

**DOI:** 10.1007/s40037-016-0272-6

**Published:** 2016-05-23

**Authors:** Jennifer Marie O’Brien

**Affiliations:** Department of Educational Administration, University of Saskatchewan, Saskatoon, SK, Canada

I read with great interest a recent article by Bourgeois et al., published in the October 2015 issue, discussing the creation of an active academic culture during residency [1]. The authors argued for the establishment of effective strategies to promote scholarly pursuits during residency, in the hopes that this will grow academically oriented clinicians. I appreciated the authors’ description of an expanded definition of scholarship that goes beyond research, and their suggestions for disseminating scholarly works that transcend traditional expectations of publishing original research.

While I respect their description of the ways in which an active scholarly culture might be encouraged (e. g. mentorship, protected time, presentation opportunities, etc.), the authors do not define their understanding of culture, nor do they describe how to gain leverage for enhancing the scholarly culture of a residency programme.

This discussion was greatly enhanced by an accompanying commentary by Chan et al. [2], which outlined how an illustrative case might be addressed through Bolman and Deal’s ‘Four Frames’ model. Chan and colleagues argued that ‘each of the frames affects the development and evolution of organizational culture, but the symbolic frame includes its most tangible features.’ I would like to draw the authors’ attention to work which both supports their argument and draws upon the symbolic frame to conceptualize the research culture in postgraduate medical education.

I identified symbolic manifestations of the research culture in one postgraduate medical education programme in anaesthesiology to raise awareness of these cultural influences on the broader research training of postgraduate trainees [3]. The case narrative was presented in two parts. First, I identified manifestations of the research culture through a symbolic frame, which shed light on the culture’s underlying values. In the second part, I theorized the application of a systems approach to a learning organization [4], highlighting the ways in which aspects of culture influence each other, and discussed techniques that might inform the work of a leader wishing to leverage the research culture in postgraduate medical education.

Identifying barriers and enablers to scholarly output is a good start; however, a more comprehensive understanding of organizational culture can leverage enhancement of the scholarly culture within residency.
